# Food addiction and its relationship with other eating behaviours among Spanish university students

**DOI:** 10.1186/s40337-023-00772-5

**Published:** 2023-04-12

**Authors:** Tamara Escrivá-Martínez, Laura Galiana, Rocío Herrero, Marta Rodríguez-Arias, Fernando Fernández-Aranda, Ashley N. Gearhardt, Rosa M. Baños

**Affiliations:** 1 Department of Personality, Evaluation and Psychological Treatment, Faculty of Psychology, University of Valencia, 46010 Valencia, Spain; 2 Polibienestar Research Institute, University of Valencia, 46022 Valencia, Spain; 3 CIBERObn Physiopathology of Obesity and Nutrition, Instituto de Salud Carlos III, 28029 Madrid, Spain; 4 Department of Methodology for the Behavioural Sciences, Faculty of Psychology, University of Valencia, 46010 Valencia, Spain; 5 Department of Psychology and Sociology, University of Zaragoza, 44003 Teruel, Spain; 6 Department of Psychobiology, Faculty of Psychology, University of Valencia, 46010 Valencia, Spain; 7 Department of Psychiatry, Bellvitge University Hospital-IDIBELL, Barcelona, Spain; 8 Department of Clinical Sciences, School of Medicine, University of Barcelona, Barcelona, Spain; 9 Department of Psychology, University of Michigan, Ann Arbor, 530 Church St., Ann Arbor, MI 48109 USA

**Keywords:** Food addiction, Modified Yale Food Addiction Scale 2.0, Psychometric properties, Spanish validation, Convergent validity, Eating styles, Binge eating, Bulimia, Body Mass Index

## Abstract

**Background:**

Food addiction (FA) is characterised by symptoms such as loss of control over food consumption, inability to reduce consumption despite the desire to do so, and continued consumption despite negative consequences. The modified Yale Food Addiction Scale 2.0 (mYFAS 2.0) is a widely used instrument to assess FA.

**Objectives:**

To validate the Spanish mYFAS 2.0; to analyse the relationships between FA with other eating behaviours, sociodemographic variables, and Body Mass Index (BMI); and to test the eating-related variables that account for the variance in FA.

**Methods:**

The sample consisted of 400 university students (*M*_*age*_ = 24.16, *SD*_*age*_ = 6.12; 51% female), who completed the mYFAS 2.0 and measures of eating-related constructs.

**Results:**

A confirmatory factor analysis (CFA) supported the one-factor structure of the mYFAS 2.0. The scale showed good internal consistency (α = .78), and good convergent validity with the mYFAS. FA was related to eating styles, binge eating, and bulimia. No differences in FA were observed between males and females, and there was no association between FA and BMI. In addition, younger participants scored higher on FA than older participants. The eating-related variables explain 54.7% of the variance in FA.

**Conclusions:**

The mYFAS 2.0 is a valid and reliable scale to assess FA in the Spanish population. The positive and significant relationship of variables related to eating (eating styles, binge eating and bulimia) with FA was demonstrated. These variables were indicated by those at high risk of FA.

## Abstract (plain english summary)

Food addiction (FA) is characterised by excessive and dysregulated intake of high-calorie foods. Loss of control over food consumption, inability to reduce consumption, and continued consumption despite negative consequences are some of its symptoms. The modified Yale Food Addiction Scale 2.0 (mYFAS 2.0) is a brief instrument specifically developed to assess FA. The objectives of this study were: (1) to examine the psychometric properties of the Spanish mYFAS 2.0; (2) to analyse the relationships between FA with other eating behaviours, sociodemographic variables, and Body Mass Index (BMI); and (3) to test whether there are certain dietary variables that may be positively related to FA. We were able to provide evidence that the mYFAS 2.0 is a valid and reliable scale for assessing FA in the Spanish population. The positive and significant relationship of eating-related variables (eating styles, binge eating and bulimia) with FA was demonstrated. These variables can be considered to identify subgroups at high risk of FA.

## Background

Food addiction (FA) refers to specific food-related behaviours characterised by excessive and dysregulated consumption of high-calorie foods [1]. Symptoms include loss of control over food consumption, inability to reduce consumption despite the desire to do so, and continued consumption despite negative consequences [2]. This concept has sparked controversy since some question its validity [3] while others support its evidence, suggesting that FA may share several characteristics with substance use disorders that may reflect common neural (e.g., brain reward pathways) [4] and psychological factors (e.g., drug/food preoccupation) [5]. In this regard, the most widely used instrument to assess FA translates the Diagnostic and Statistical Manual of Mental Disorders (DSM) criteria for substance use disorders to eating behaviour. Specifically, the original Yale Food Addiction Scale (YFAS) [6] translated the DSM-IV-TR diagnostic criteria for substance dependence [7], and the new version of this instrument (YFAS 2.0; [8]) reflects the changes in the DSM-5 diagnostic criteria for substance use disorder [9]. There is a short version (mYFAS 2.0; [10]) which has demonstrated good psychometric properties and an underlying one-factor structure [10]. The mYFAS 2.0 has been adapted to some languages (Italian, Portuguese/Brazilian, French, Arabic, Chinese, or Czech) [11–17], in which it has been traditionally studied as a unidimensional construct, but has not yet been adapted to Spanish.

Recently, interest in FA research has been growing, especially because it has been associated with higher rates of eating disorders (in particular, binge eating disorder), and overweight or obesity [1, 18]. Some data point out that around 60–80% of people with binge eating disorder report clinical symptoms of FA [19, 20], and 25% of people with obesity meet significant criteria for FA [21, 22]. In addition, the literature indicates that FA can also be present in healthy weight individuals with no history of eating disorders, with some studies indicating 11% of healthy weight individuals reporting these symptoms [21, 22].

Research on the prevalence of FA in non-clinical individuals has examined the role of age and gender, with contradictory results. The most supported hypothesis is that young people are more likely to show FA than older individuals [10, 23], and this could be explained because the stage of youth involves many physiological and psychological changes that may increase vulnerability to environmental threats (such as addictions or inappropriate eating habits) [24]. However, one study has found an increased likelihood of FA in adulthood [22], suggesting that older people are more exposed to unhealthy foods, and this repeated exposure may reduce the sensitivity of the brain reward circuitry, which could lead them to consume more food to feel pleasure [25].

In relation to gender, the results are contradictory as well, with some studies supporting higher FA in women [8, 22, 26, 27], while others (including a meta-analysis) find no differences [10, 28, 29]. A recent meta-analysis supports higher FA in men [30], although the study samples were predominantly women, which limits the generalisability of the results. Given the high prevalence of FA in the non-clinical sample and the inconsistency of the results, further research is necessary to identify vulnerable individuals in order to better design the prevention and treatment of this problem.

Research has also been interested in identifying which factors may be related to FA. Among other implicated variables [31], several studies point to a relationship between FA and eating-related variables, suggesting that some eating behaviours and eating styles could be related to this problematic food intake. For example, FA has been linked to emotional eating [10], external eating [32], restrained eating [33], binge eating [34], bulimia [35], and Body Mass Index (BMI) [10]. These eating behaviours are largely related to the consumption of unhealthy foods, and such repeated consumption over time may be related to FA. It is important to understand the specific relationships between all these variables in order to better understand the FA problem, especially in young people.

The FA construct seems to share clinical characteristics with other types of addictions, and the prevalence is generally high, especially in young people. However, studies in the Spanish population are scarce. Moreover, to date, the prevalence of FA and its relationship with sociodemographic characteristics and eating-related variables in young Spanish students is unknown. It is essential to evaluate the co-existence of this problem with other eating problems in this population, as this may help to define, understand, and treat this problem in this young population.

Hence, this study has several objectives. First, it aims to examine the psychometric properties of the Spanish translation of mYFAS 2.0 in a sample of young Spaniards from a university setting by assessing the factorial structure through structural equation modelling, specifically confirmatory factor analysis (CFA), and providing evidence of its internal consistency. This study also aims to analyse the relationship of FA with other eating-related behaviours (emotional eating, external eating, restrained eating, binge eating, and bulimia), with sociodemographic characteristics (age, gender), and BMI. Finally, this study will test the direct relationship of eating variables related to FA (emotional eating, external eating, restrained eating, binge eating, and bulimia) through structural equation modelling.

## Methods

### Participants

The sample consisted of college students from the province of Valencia (Spain). A total of 400 participants took part in the study, 204 females (51%; mean age 23.35 ± 4.65) and 196 males (49%; mean age 24.99 ± 7.26).

According to the World Health Organisation [36], 24 participants (6%) were underweight (BMI < 18.5), 296 (74%) had healthy weight (18.5 ≥ BMI ≤ 24.99), 64 (16%) were overweight (25 ≥ BMI ≤ 29.99), and 15 (3.8%) were obese (BMI ≥ 30). One participant did not respond. Female students had a mean BMI of 21.90 (SD = 3.22), and male students had a mean BMI of 23.87 (SD = 4.23).

### Measures

The study included the collection of sociodemographic information (i.e., gender, with two categories: female and male, and age). Body Mass Index (BMI) was calculated by dividing self-reported current weight by height squared (kilograms/metres^2^) [36]. The questionnaires used in the study were:

### Modified Yale Food Addiction Scale 2.0

The mYFAS 2.0 is a self-report questionnaire that measures FA [10]. It is the short form of the YFAS scale 2.0 [8]. It consists of 13 items presented on an 8-point Likert-type scale (0 = never, 7 = every day). The scale specifically measures addictive eating behaviours during the past year through the first 11 items (e.g., “I avoided work, school, or social activities because I was afraid of overeating there.”, “My overeating prevented me from taking care of my family or doing household chores.”). The last two items (items 12 and 13) assess clinical significance, i.e., clinically significant impairment or distress (see “Appendix”).

The mYFAS 2.0 offers two scoring options: a symptom count and a diagnostic score based on DSM-5 criteria for substance use disorder. For the symptom count scoring option, each item from 1 to 11 is scored 0 or 1 depending on whether it meets the established criteria as determined by the original validation (0 = does not meet criteria; 1 = meets criteria). Finally, the item scores are summed and a score between 0 and 11 (0 to 11 symptoms) is obtained; clinical significance (the last two items) is not added to the score. For cut-off values and the assessment procedure, see Zhang et al. [15]. For the diagnostic scoring option, the symptom count score is obtained, and the clinical significance criterion (impairment or distress) is also added, obtaining a score that divides the sample as follows: does not meet FA criteria (one or fewer symptoms and/or does not meet impairment or distress), mild FA (two to three symptoms plus impairment or distress), moderate FA (four or five symptoms plus impairment or distress), or severe FA (six or more symptoms plus impairment or distress) (Note: in the present article, we talk about “diagnostic scores” when we use the “diagnostic scoring option).

The Spanish translation of this questionnaire underwent a rigorous procedure. First, the questionnaire was translated from English into Spanish by a bilingual translator. Second, three Spanish reviewers familiar with the study reviewed the translated items. Third, the study researchers evaluated the scale for correct understanding and administered it to 20 students to corroborate that it was understandable. The Spanish version was an exact translation of the original version [10]. The final version of the English and Spanish mYFAS 2.0 and its instructions are available in “Appendix”. Internal consistency in this study is reported in the Results section.

### Modified Yale Food Addiction Scale

The mYFAS is a brief 9-item scale that measures FA [37]. It was developed from the YFAS scale [6]. Both scales were developed following the diagnostic criteria for substance use according to the DSM-IV-R. This scale consists of 9 items, of which the first 7 measure diagnostic criteria and the last two items assess the presence of clinically significant impairment and distress. Estimated with Cronbach's alpha, the internal consistency in this sample was 0.77. The same translation procedure for the mYFAS 2.0 scale was used.

### Dutch Eating Behaviour Questionnaire

The Dutch Eating Behaviour Questionnaire (DEBQ; [38]) is a self-report instrument composed of 33 items that assess three types of eating behaviour: 13 items for emotional eating (eating in response to negative emotions, such as anger or anxiety), 10 items for external eating (eating in response to external food-related stimuli), and 10 items for restrained eating (voluntary restriction of eating to reduce or maintain weight). It uses a five-point Likert-type scale (1 = never, 5 = very often). In this study, we used the Spanish version [39]. The Cronbach's alphas for this study were for emotional eating: 0.95, for external eating: 0.87, and for restrained eating: 0.90.

### Binge Eating Scale

The Binge Eating Scale (BES; [40]) is a self-administered questionnaire composed of 16 items that manifest behavioural and cognitive disturbances regarding food (e.g., eating large amounts of food or worrying about food). Items are answered on a 4-point Likert-type scale (0 = no severe binge eating symptoms; 3 = severe binge eating symptoms). The scores range from 0 to 46, with higher scores indicating binge eating severity. For the present study, the Spanish validation of the BES was used [41]. The Cronbach's alpha for the present sample was 0.87.

### EDI-3 bulimia subscale

In this study, the bulimia subscale of the EDI-3-RF was used [42, 43]. This subscale consists of 8 items measuring bulimia risk (e.g., “I think about vomiting to lose weight,”: “I tend to binge eat”). The items are rated on a 6-point Likert scale (1 = always, 6 = never). The Cronbach's alpha for this sample was 0.84.

### Design and procedure

A cross-sectional design was used in the present study with one-time point data collection. Data were collected in June 2022. Participants were invited to take part by the announcement of the study in the classrooms at the University of Valencia and online. They were informed that they had to complete questionnaires related to eating-related variables. All the surveys were administered through the Lime Survey Platform. Participants did not receive financial compensation or any other type of incentive for their participation.

### Ethical considerations

The study was conducted following the ethical standards of the Declaration of Helsinki in 1964 and was approved by the Ethics Committee of the University of Valencia (Registration number: H151385403893939). All participants were informed of the confidentiality of the data and voluntarily collaborated in the study. All of them gave their informed consent prior to their inclusion in this study.

### Statistical analysis

Analysis included a CFA, which hypothesised a one-factor structure, for the symptom count score of the 11 items from the mYFAS 2.0. Items included in the analysis were items from 1 to 11. Items 12 and 13 were excluded as they are only used for clinical significance. The hypothesised internal structure was based on previous evidence gathered by Schulte and Gearhardt [10], as well as other translations of the scale which demonstrated a unidimensional structure [11–14]. Weighted Least Square Mean and Variance Adjusted Estimators (WLSMV) was used, given the multivariate non-normality of the data. In order to assess model fit, the fit criteria used were: the chi-square, the Comparative Fit Index (CFI), and the Root Mean Square Error of Approximation (RMSEA). CFI above 0.90 (better if above 0.95) and RMSEA below 0.08 (better if below 0.05) indicate good fit [44].

Internal consistency estimates were also calculated and included both Cronbach's alpha and Omega for the symptom count score of the mYFAS 2.0. Once the internal structure and consistency were studied, descriptive statistics for the scale were calculated, using means and standard deviations for the symptom count scores and for the diagnostic threshold scoring method.

To test for convergent validity, a CFA was estimated including the symptom count scores. Traditionally, convergent validity is defined as the extent to which responses on a test exhibit a strong relationship with responses on conceptually similar tests. Indeed, the previous version of a test is the most similar version. Therefore, a general factor of FA explaining the 11 items of the mYFAS 2.0 together with the 7 items of the mYFAS was hypothesised. mYFAS was included to test for convergent validity. As both versions of the scale measure the same construct, FA, for evidence of convergent validity, good general and analytical fit is expected, meaning a general factor of FA would explain the items of the two scales.

To analyse potential differences and relationships between mYFAS 2.0 and age, gender, and BMI, we applied the chi square test, t-test, analysis of variance (ANOVA), and Pearson correlations. Additionally, the relationships between FA (measured with the mYFAS 2.0 symptom count scores) and emotional eating, external eating, restrained eating, binge eating, and bulimia were analysed using Pearson correlations and ANOVAs.

Finally, we tested the predictive power of the variables related to FA in a structural equation modelling context. For this purpose, a structural equation model was hypothesised, estimated, and tested, in which the aforementioned variables and their relationship with FA were simultaneously tested, using symptom count scores. Specifically, a multiple indicators and multiple causes (MIMIC) model was computed, modelling FA as the single latent variable. Weighted Least Square Mean and Variance Adjusted Estimators (WLSMV) was used. To assess model fit, we used the fit criteria reported before. All the analysis were performed using IBM SPSS Statistics for Windows, Version 24.0 [45] and Mplus, Version 8 [46].

## Results

### Modified Yale Food Addiction Scale 2.0 factor structure and internal consistency

A CFA, based on the structure found by Schulte and Gearhardt [10], with a one-factor solution was conducted with the first 11 items of the mYFAS 2.0. The model showed an excellent fit: *χ*^*2*^(44) = 59.34 (*p* = 0.061); CFI = 0.99; RMSEA = 0.03 [0.000, 0.047]. Factor loadings were statistically significant (*p* < 0.001), with values of 0.58 or higher (see Table 1).

**Table 1:** Descriptive statistics and factor loadings for mYFAS 2.0 symptom count scores

Item number	Item content	Symptom count scores		
		M	SD	λ
1	I ate to the point where I felt physically ill	0.46	0.49	.64
2	I tried and failed to cut down on or stop eating certain foods	0.48	0.50	.75
3	I spent more time feeling sluggish or tired from overeating	0.63	0.48	.66
4	I avoided work, school or social activities because I was afraid I would overeat there	0.10	0.30	.58
5	I kept eating in the same way even though my eating caused emotional problems	0.27	0.44	.80
6	Eating the same amount of food did not give me as much enjoyment as it used to	0.31	0.46	.74
7	If I had emotional problems because I had not eaten certain foods, I would eat those foods to feel better	0.46	0.49	.59
8	My friends or family were worried about how much I overate	0.14	0.35	.75
9	My overeating got in the way of me taking care of my family or doing household chores	0.04	0.20	.99
10	I was so distracted by eating that I could have been hurt (e.g. when driving a car, crossing the street and operating machinery)	0.09	0.29	.69
11	I had such strong urges to eat certain foods that I could not think of anything else	0.55	0.49	.64

Regarding the internal consistency of the mYFAS 2.0, the scale showed good internal consistency. When internal consistency estimates were calculated, Cronbach's alpha was 0.78 and Omega was 0.92. The mean using symptom count scoring method was 3.58 (SD = 2.62), with a minimum core of 0 and a maximum score of 11. Using the diagnostic threshold scoring method, 12.5% met mild FA (n = 50); 11.3% met moderate FA (n = 45); and 20.6% met severe FA. They were considered to have a high probability of FA if they had more than three symptoms plus impairment or distress. Thus, 31.9% were at risk for meeting FA criteria.

**Fig. 1:**
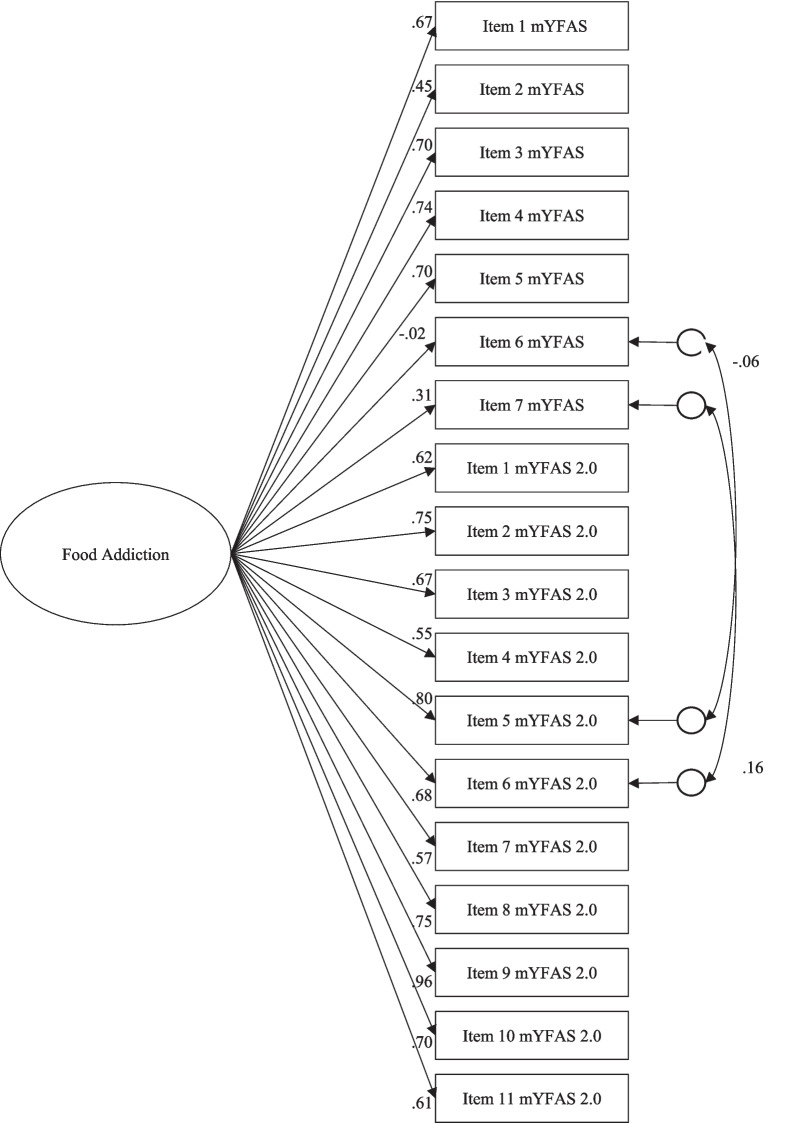
Results of the confirmatory factor analysis, including mYFAS 2.0 and mYFAS scores. *Notes*: All factor loadings were statistically significant (*p* < .001), except for Item 8 mYFAS. The residual correlations were not statistically significant (*p* > .050)

Convergent validity was studied using the mYFAS. For this purpose and considering that both mYFAS 2.0 and mYFAS assess FA, a CFA in which one factor of FA explained the 9 items of the mYFAS 2.0 together with the 7 items of the mYFAS was conducted for the symptom count scores of the two measures. In order to avoid multi-collinarity problems, residuals of items with the same content and similar wording were estimated. These included correlations for the mYFAS item 6 and mYFAS 2.0 item 5, and mYFAS item 7 and mYFAS 2.0 item 6 (for mYFAS items, see Meule and Gearhardt, [47]; for mYFAS 2.0 items, see Table 1). The model showed adequate fit: *χ*^*2*^(133) = 240.80 (*p* = 0.001); CFI = 0.96; RMSEA = 0.05 [0.036,0.054]. As shown in Fig. 1, all factor loadings were statistically significant except for item 8 of the mYFAS, which showed a non-statistically significant result. Residual correlations were not statistically significant. This demonstrates convergent validity for the mYFAS 2.0, meaning the new items are also a measure of FA, as the ones of the original version (mYFAS) were. There is only one factor, FA, which adequately explained both the items in the original version and the new version.

### FA measures with demographics and BMI

Regarding age, correlations were used to study the relationship with the mYFAS 2.0. When studied with the symptom count score, the relationship was negative and statistically significant (*r* = − 0.13, *p* = 0.011; and *r* = − 0.16, *p* = 0.001; respectively), with younger individuals reporting higher addictive-like eating behaviours than older participants (Table 2). No significant association was found between age and the diagnostic scoring method: *F*(3, 395) = 1.68, *p* = 0.171, *η*^*2*^ = 0.01 (see Table 3).

**Table 2:** Correlations between mYFAS 2.0 symptom count scores, emotional eating, external eating, restrained eating, binge eating, bulimia, age, and BMI

	mYFAS 2.0 symptom count
Emotional eating	r = .59 (*p* < .001)
External eating	r = .43 (*p* < .001)
Restrained eating	r = .40 (*p* < .001)
Binge eating	r = .65 (*p* < .001)
Bulimia	r = .59 (*p* < .001)
Age	r = − .16 (*p* = .001)
BMI	r = .08 (*p* = .137)

**Table 3:** Descriptive statistics for emotional eating, external eating, restrained eating, binge eating, bulimia, age, and BMI, for the different FA diagnostic scores

	No food addiction		Mild food addiction		Moderate food addiction		Severe food addiction		*p* value
	M	SD	M	SD	M	SD	M	SD	
Emotional eating	22.44	8.11	26.64	8.56	29.22	10.06	38.27	12.15	< .001
External eating	28.77	7.04	29.36	6.24	30.36	6.97	35.13	7.44	< .001
Restrained eating	19.77	6.75	24.92	9.13	24.20	7.71	28.15	7.77	< .001
Binge eating	3.90	3.27	5.84	4.53	7.62	5.32	14.54	7.64	< .001
Bulimia	1.12	2.08	1.42	1.90	3.09	3.67	6.63	6.33	< .001
Age	24.60	6.46	24.78	7.80	23.24	3.45	23.12	5.00	.171
BMI	22.55	3.71	22.73	3.85	22.86	3.07	23.82	4.59	.088

As for gender, there were no significant differences between males and females for the symptom count: *t*(397) = − 0.62, *p* = 0.539; or diagnostic score: *χ*^*2*^(3) = 0.35, *p* = 0.950, *V* de Cramer = 0.03. Concerning BMI, there was no significant association with the symptom count score (*r* = 0.08, *p* = 0.133; and *r* = 0.08, *p* = 0.137) (Table 2). In addition, no significant association with mYFAS 2.0 diagnostic categories was found: *F*(3, 395) = 2.20, *p* = 0.088, *η*^*2*^ = 0.02 (Table 3).

### Associations among FA and other eating-related variables

FA was related to emotional eating, external eating, restrained eating, binge eating, and bulimia. Although all correlations were statistically significant (*p* < 0.001), the strongest association was with binge eating (*r* = 0.65) and the weakest was with restrained eating (*r* = 0.40) (Table 2).

When using the diagnostic scores, one-way ANOVA between subjects was used. Significant differences in emotional eating were also found: *F*(3, 395) = 58.12, *p* < 0.001, *η*^*2*^ = 0.31, with post hoc differences between those showing no FA and the rest of the groups (*p* < 0.050), with lower emotional eating for the former; also between those with severe FA and the rest of the groups (*p* < 0.001), being this the group with higher levels of emotional eating; however, no significant differences in emotional eating were found between those with mild and moderate FA (*p* > 0.050). Similar results were found for external eating, *F*(3, 395) = 16.79, *p* < 0.001, *η*^*2*^ = 0.11, although the relationship found was of smaller value. Significant post hoc differences were found between participants with severe FA and the rest of the subgroups (*p* < 0.010), with higher levels of external eating for those with severe FA. Regarding restrained eating, there were also significant differences between mYFAS 2.0 diagnostic scores: *F*(3,395) = 28.58, *p* < 0.001, *η*^*2*^ = 0.18. Post hoc comparisons pointed statistically significant differences between no FA and mild FA (*p* < 0.001), moderate FA (*p* = 0.002) and severe FA (*p* < 0.001), with lower restrained levels for those with no FA; and statistically significant differences between severe FA and mild FA, with higher levels of restrained for the former (*p* = 0.022) (see Table 3).

Additionally, the relationship of FA and binge eating scoring was studied. Binge eating showed the strongest association with FA: *F*(3, 395) = 96.63, *p* < 0.001, *η*^*2*^ = 0.42, with statistically significant post hoc differences between participants with no FA and those with moderate (*p* < 0.001) or severe (*p* < 0.001) FA; also, between mild FA and severe FA (*p* < 0.001); and between moderate FA and severe FA (*p* < 0.001). In all the cases, the higher the level of FA was, the higher the binge eating scores were (Table 3).

Finally, the relationship between FA and bulimia scoring was studied. This relationship was also found when using the mYFAS 2.0 diagnostic scores: *F*(3,395) = 50.19, *p* < 0.001, *η*^2^ = 0.28. Post hoc tests showed statistically significant differences in bulimia scores between those with no FA and those with moderate (*p* = 0.004) and severe (*p* < 0.001) FA; those with mild and severe FA (*p* < 0.001); and those with moderate and severe FA (*p* < 0.001), with higher levels of bulimia for higher FA. Please, see details in Table 3.

### Structural equation modelling predicting FA

**Fig. 2:**
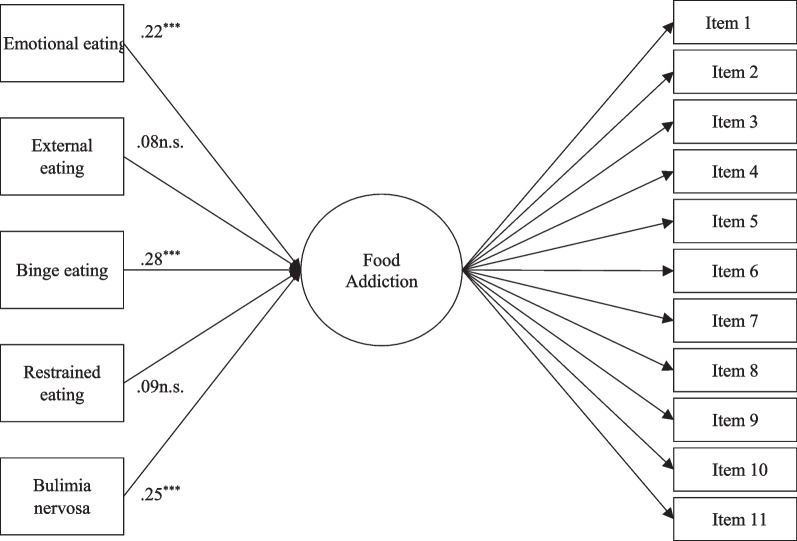
Results of MIMIC models predicting food addiction. *Notes*: *** *p* < .001; n.s. non-statistically significant. Correlations between exogenous variables can be consulted in Table 4

To test the predictive power of the variables related to FA in a multivariate context, a structural equation model was hypothesised, estimated, and tested, in which the aforementioned variables and their relationship to FA were simultaneously tested. All these variables were hypothesised to directly impact FA, as presented in Fig. 2. These variables were interrelated, with estimated covariances in the structural model among all the exogenous variables with significant product–moment correlations. Correlations are provided in Table 4.

**Table 4:** Correlations between exogenous variables in the structural equation models

	Emotional eating	External eating	Restrained eating	Binge eating
Emotional eating	–			
External eating	r = .51	–		
Restrained eating	r = .42	r = .22	–	
Binge eating	r = .64	r = .48	r = .54	–
Bulimia	r = .65	r = .47	r = .35	r = .74

All correlations were statistically significant (*p* < .001)

The model (Fig. 2) fitted the data excellent: *χ*^*2*^(94) = 169.12 (*p* < 0.001); CFI = 0.97; RMSEA = 0.05 [0.034, 0.055]. All factor loadings were statistically significant (*p* < 0.001). External eating and restrained eating showed no statistically significant predictive power over FA, and, again, binge eating showed the strongest relation. In all, 54.7% of FA variance was explained (*R*^*2*^ = 0.55, *p* < 0.001).

## Discussion

The aim of this study was to examine the psychometric properties of the Spanish version of the mYFAS 2.0 in a sample of young Spaniards from a university setting, to analyse the relationships between FA with other eating behaviours, sociodemographic variables and BMI, and to test the predictive power of eating behaviour variables related to FA using structural equation modelling.

The factor structure of the scale was assessed using the CFA. The scale provided evidence of a single dimension of FA, which adequately explained all 11 mYFAS 2.0 items. This result is consistent with Schulte and Gearhardt [10], who also suggested a good fit for a single-factor model (CFI ≥ 0.95; TLI ≥ 0.95). In addition, all items in the original validation had factor loadings for a single factor of 0.58 or higher. Therefore, given the goodness-of-fit parameters and good factor loadings for each item, the authors maintained a single-factor solution for the mYFAS 2.0 [10]. As for the internal consistency of the mYFAS 2.0, Cronbach's alpha and Omega were calculated using its recoded version for symptom count. The results were good, pointing to the accuracy of the instrument for assessing FA in the Spanish population.

In our study, the frequency of FA revealed that 31.9% of participants were at risk for meeting FA criteria. This frequency is higher than that described in the Granero et al. [48] study, who reported 3.3% FA in young Spanish women using YFAS 2.0. Other studies conducted with university samples showed higher prevalence, ranging from 11.4 to 25% [6, 49, 50]. A recent meta-analysis pointed out that the prevalence of FA in non-clinical samples is 14% [30]. Beyond the possible differences that may arise from the use of different versions of YFAS2.0 (we used mYFAS2.0), the frequency of FA in our study is high and may be related to the recruitment methodology; for instance, when participants were recruited, they were informed to “fill out questionnaires related to diet-related variables,” which may have marked a tendency for participants with more eating problems to respond. This result points to the need to carry out an in-depth analysis on the role of specific variables (age, sex, general psychopathology, including eating disorders, and BMI, etc.) to have a clearer understanding of their FA presence in the general young population.

The present study also analysed the adequacy of a general factor of FA for both versions, the mYFAS 2.0 and the mYFAS. Results of CFA pointed out that both scales measure, indeed, the same construct. The models were expected to fit adequately, as both scales have good psychometric properties, and the symptoms and scoring form are very similar [10, 51]. This is the first time that the relationship between the two measures has been studied.

This study has also analysed the association between FA and sociodemographic variables (age and gender). In relation to age, higher FA scores were observed in younger participants. Previous studies have also found that younger individuals score higher on FA symptomatology than older individuals [10, 23]. This could be seen as part of a higher vulnerability to addiction in younger people, who can be more susceptible to environmental effects. Their ability to assess risks can be more limited and they may have more difficulties in controlling their resistance of the consumption of high fat/sugar foods. However, we cannot forget that most of our sample was young, so these results should be interpreted with caution given the low variability of age in the data.

With regard to gender, no significant differences were observed. There is evidence confirming no relationship between addictive-type eating and gender [10, 23, 28]. However, some studies have found that women reported a higher number of FA symptoms than men [8, 22], although these studies are usually composed of a population with eating disorders [30] or women who are overweight or obese [22]. Our participants are young people of both sexes, with a normal BMI distribution, so this sample composition could explain the absence of differences.

Regarding BMI, no significant correlation with FA was observed. The original study by Schulte and Gearhardt [10] found a relationship, although the effect size was small. Other subsequent studies with non-clinical samples also did not find this relationship [28, 52]. Again, the characteristics of the samples could explain these differences, and specifically the BMI scores. Participants from the original study showed a high BMI (mean about 27 points), whereas our sample showed a lower BMI (mean 23 points). In addition, this may be because individuals have a tendency to gain weight as they age, and poor dietary habits cannot yet be demonstrated in weight gain at this age. In the future, it is important to test whether FA predicts future weight gain in the high-risk periods of late adulthood.

The relationships between FA and other relevant eating behaviour variables (emotional eating, external eating, restrained eating, binge eating, and bulimia) were analysed. The mYFAS 2.0 scale was found to be related to all of them. The results for emotional and external eating are in line with previous literature [6, 53]. A greater tendency to eat in order to cope with emotional stressors (e.g., anxiety or depression) and/or environmental triggers (e.g., in the presence of palatable food) and its relationship with FA is well known in the existing literature. Emotional eaters tend to eat more and more sweet foods after being in a negative mood [54], suggesting that food can play a role in mitigating negative mental states, which may lead to increased food consumption in order to feel better [55], and this may perhaps contribute to FA. Similarly, external eaters tend to report higher energy intake [56]. This overeating is related to a reduced brain reward system response [25], which may be related to the consumption of larger amounts of food to feel good.

Our data also show a positive relationship between restrained eating and FA in accordance with previous studies [33, 57]. This relationship might be mediated by episodic binge eating, since it has been suggested that dieting can precipitate binge eating [33, 57]. However, this association could come from the reverse direction, in the sense that young people who exhibit FA behaviours may try to restrict eating to maintain or lose weight. Future non-correlational studies are needed to clarify how this relationship between dietary intake restriction and FA is established.

Regarding binge eating, a strong positive relationship with FA was observed, demonstrating the strongest relationship among all variables in the same direction as previous studies [8, 41, 58]. It could be speculated that elevated food consumption may sensitise the dopamine-mesolimbic reward system, resulting in an excessive increase in wanting to continue consuming food, as occurs with drugs [59]. In line with this result, a positive relationship between bulimia and FA was also observed. Meule et al. [60] also noted that bulimia was positively and strongly associated with FA. This relationship could be because it has been investigated that 100% of the foods consumed in binge episodes in eating disorders populations (e.g., bulimia) are the types of ultra-processed foods that are considered to be addictive [61], which may contribute to the association between these constructs.

Finally, our last objective was to analyse the statistical predictive power of the eating-related variables related to FA (emotional eating, external eating, restrained eating, binge eating, and bulimia) using structural equation modelling. A positive and significant relationship of emotional eating with FA was observed. However, it is noteworthy that both external and restrained eating did not explain part of the variance in FA. These results open the way for future studies to carry out ecological momentary assessments to evaluate whether these eating styles can predict FA. All these studies can help us to adapt and improve programmes focused on FA prevention.

Another relevant finding is the high relationship between binge eating and FA. In this line, bulimia was also positively and significantly related to FA. The pattern of bulimia (binge eating and food restriction) shares many neurobiological characteristics with drug addiction, for example, the dopaminergic, glutamatergic, and opioid systems are known to play similar roles in both [62]. It would be desirable for future studies to corroborate through longitudinal and experimental designs to explore whether binge eating and /or bulimia can act as risk factors for FA.

Our findings revealed a close association between FA and eating behaviour variables. To the best of our knowledge, this is the first study to demonstrate the relationship of all these dietary variables with FA, which points to the relevance of these variables in explaining the variance of FA.

Some limitations of the study should be noted. First, self-reported height and weight were used to calculate BMI. These data should be viewed with caution because weight tends to be underestimated and height to be overestimated [63]. Second, since these are university students, the results cannot be generalised to other populations or to a clinical setting. Third, the data were cross-sectional, so no firm conclusions can be drawn about the direction of the associations obtained. More longitudinal designed studies with large and heterogeneous samples are needed to explore these relationships in depth. Fourth, and lastly, there are other variables that were not evaluated and may be relevant, including ethnic and socioeconomic characteristics, the diagnosis of eating disorders, psychopathology, and personality traits.

## Conclusions

In conclusion, the Spanish version of the mYFAS 2.0 showed adequate psychometric properties to assess FA in a sample of young Spaniards. Studies could benefit from this scale to identify participants with FA. Furthermore, the positive and significant relationship of eating-related variables with FA (emotional eating, external eating, restrained eating, binge eating and bulimia) was demonstrated. This is the first study to point to the power of eating-related variables in explaining part of the variance in FA, together explaining 54.7% of the variance in FA. All these variables can be considered to identify subgroups at high risk for FA.

## Data Availability

The datasets used and/or analysed during the current study are available from the corresponding author on reasonable request.

## Appendix

### English version of the mYFAS 2.0 scale

#### Modified Yale Food Addiction Scale version 2.0 (mYFAS 2.0)

This survey asks about your eating habits in the past year. People sometimes have difficulty controlling how much they eat of certain foods such as:

- Sweets like ice cream, chocolate, doughnuts, cookies, cake, candy
- Starches like white bread, rolls, pasta, and rice
- Salty snacks like chips, pretzels, and crackers
- Fatty foods like steak, bacon, hamburgers, cheeseburgers, pizza, and French fries
- Sugary drinks like soda pop, lemonade, sports drinks, and energy drinks

When the following questions ask about “CERTAIN FOODS” please think of ANY foods or beverages similar to those listed in the food or beverage groups above or ANY OTHER foods you have had difficulty with in the past year.

**Table:**

In the past 12 months	Never	Less than monthly	Once a month	2–3 times a month	One a week	2–3 times a week	4–6 times a week	Every day
1. I ate to the point where I felt physically ill	0	1	2	3	4	5	6	7
2. I tried and failed to cut down on or stop eating certain foods	0	1	2	3	4	5	6	7
3. I spent a lot of time feeling sluggish or tired from overeating	0	1	2	3	4	5	6	7
4. I avoided work, school or social activities because I was afraid I would overeat there	0	1	2	3	4	5	6	7
5. I kept eating in the same way even though my eating caused emotional problems	0	1	2	3	4	5	6	7
6. Eating the same amount of food did not give me as much enjoyment as it used to	0	1	2	3	4	5	6	7
7. If I had emotional problems because I hadn’t eaten certain foods, I would eat those foods to feel better	0	1	2	3	4	5	6	7
8. My friends or family were worried about how much I overate	0	1	2	3	4	5	6	7
9. My overeating got in the way of me taking care of my family or doing household chores	0	1	2	3	4	5	6	7
10. I was so distracted by eating that I could have been hurt (e.g., when driving a car, crossing the street, operating machinery)	0	1	2	3	4	5	6	7
11. I had such strong urges to eat certain foods that I couldn’t think of anything else	0	1	2	3	4	5	6	7
12. I had significant problems in my life because of food and eating. These may have been problems with my daily routine, work, school, friends, family, or health	0	1	2	3	4	5	6	7
13. My eating behavior caused me a lot of distress	0	1	2	3	4	5	6	7

## Spanish version of the mYFAS 2.0 scale

### Escala Yale de adicción a los alimentos modificada versión 2.0 (mYFAS 2.0)

Esta encuesta pregunta sobre tus hábitos alimentarios del año pasado. Las personas a veces tienen dificultad para controlar la cantidad que comen de ciertos alimentos, como, por ejemplo:

- Dulces como helado, chocolate, donuts, galletas, pastel, caramelos
- Almidones como pan blanco, panecillos, pasta y arroz
- Aperitivos salados como papas fritas, rosquilletas y galletas saladas
- Alimentos ricos en grasa como bistec, tocino, hamburguesas, pizza y papas fritas
- Bebidas azucaradas como gaseosas, refrescos, bebidas para deportistas y bebidas energéticas

Cuando las siguientes preguntas se refieran a “DETERMINADOS ALIMENTOS”, por favor piensa en CUALQUIER alimento o bebida similar a los enumerados en los grupos de alimentos o bebidas anteriores o CUALQUIER OTRO alimento con el que hayas tenido dificultad en el último año.

**Table:**

En Los Últimos 12 Meses	Nunca	Menos de una vez al mes	Una vez al mes	2–3 veces al mes	Una vez a la semana	2–3 veces a la semana	4–6 veces a la semana	A diario
1. He comido hasta el punto de sentirme físicamente enfermo	0	1	2	3	4	5	6	7
2. Lo intenté y no pude reducir o dejar de comer ciertos alimentos	0	1	2	3	4	5	6	7
3. He pasado mucho tiempo sintiéndome lento o cansado por comer en exceso	0	1	2	3	4	5	6	7
4. He evitado el trabajo, la escuela o las actividades sociales porque tenía miedo de comer en exceso allí	0	1	2	3	4	5	6	7
5. Seguí comiendo de la misma manera a pesar de que mi alimentación me ha causado problemas emocionales	0	1	2	3	4	5	6	7
6. Comer la misma cantidad de comida no me ha dado tanto placer como solía hacerlo	0	1	2	3	4	5	6	7
7. Si tuviera problemas emocionales por no comer ciertos alimentos, los comería para sentirme mejor	0	1	2	3	4	5	6	7
8. Mis amigos y familiares estaban preocupados por lo mucho que yo comía	0	1	2	3	4	5	6	7
9. Mi exceso de comida me impidió cuidar de mi familia o hacer tareas domésticas	0	1	2	3	4	5	6	7
10. Estaba tan distraído al comer que podría haberme herido (por ejemplo, al conducir un coche, al cruzar la calle o al operar con maquinaria)	0	1	2	3	4	5	6	7
11. Tenía tantas ganas de comer ciertos alimentos que no podía pensar en otra cosa	0	1	2	3	4	5	6	7
12. Tuve problemas significativos en mi vida debido a la comida y al comer. Estos pueden haber sido problemas con mi rutina diaria, trabajo, escuela, amigos, familia, o salud	0	1	2	3	4	5	6	7
13. Mi comportamiento alimentario me ha causado mucho malestar	0	1	2	3	4	5	6	7

## Abbreviations

ANOVA: Analysis of variance
BES: Binge Eating Scale
BMI: Body Mass Index
CFA: Confirmatory factor analysis
CFI: Comparative Fit Index
DSM: Diagnostic and statistical manual of mental disorders
FA: Food addiction
MIMIC: Multiple indicators and multiple causes
mYFAS: Modified Yale Food Addiction Scale
mYFAS 2.0: Modified Yale Food Addiction Scale 2.0
RMSEA: Root mean square error of approximation
WLSMV: Weighted least square mean and variance adjusted estimators
YFAS: Yale Food Addiction Scale
